# Effects of Obesity on Pro-Oxidative Conditions and DNA Damage in Liver of DMBA-Induced Mammary Carcinogenesis Models

**DOI:** 10.3390/metabo7020026

**Published:** 2017-06-08

**Authors:** Stepan Melnyk, Soheila Korourian, Joseph W. Levy, Oleksandra Pavliv, Teresa Evans, Reza Hakkak

**Affiliations:** 1 Department of Pediatrics, University of Arkansas for Medical Sciences, 4301 W. Markham St., Little Rock, AR 72205, USA; MelnykStepanB@uams.edu (S.M.); JWLevy@uams.edu (J.W.L.); PavlivOleksandra@uams.edu (O.P.); TTEvans@uams.edu (T.E.); 2 Arkansas Children’s Research Institute, 13 Children’s Way, Little Rock, AR 72202, USA; 3 Department of Pathology, University of Arkansas for Medical Sciences, 4301 W. Markham St., Little Rock, AR 72205, USA; Korouriansoheila@uams.edu; 4 Department of Dietetics and Nutrition, University of Arkansas for Medical Sciences, 4301 W. Markham St., Little Rock, AR 72205, USA

**Keywords:** obesity, liver, oxidative stress, DNA damage

## Abstract

The prevalence of the overweight and obesity is on the rise worldwide. Obesity can increase the risk of certain cancers and liver steatosis development. Previously, we reported that obesity increased liver steatosis in a mammary tumor model, but little is known about the effects of obesity in the liver in regard to global DNA methylation, DNA damage, and oxidative/nitrosative stress. Using a mammary tumor model, we investigated the effects of obesity on oxidative stress and DNA reaction. Five-week-old lean and obese female rats were used. At 50 days of age, all rats received 7,12-dimethylbenz(*α*)anthracene (DMBA) and were sacrificed 155 days later. HPLC with electrochemical and ultraviolet detection and LC-MS were used. Obesity caused higher (*p* < 0.0004) methionine levels, had no effect (*p* < 0.055) on SAM levels, caused lower (*p* < 0.0005) SAH levels, caused higher (*p* < 0.0005) SAM/SAH ratios, and increased (*p* < 0.02) global DNA methylation. Levels of free reduced GSH were not significantly lower (*p* < 0.08), but free oxidized GSSG was higher (*p* < 0.002) in obese rats. The GSH/GSSG ratio was lower (*p* < 0.0001), and oxidized guanosine was higher (*p* < 0.002) in DNA of obese rats compared to lean rats. Obesity caused significant oxidative/nitrosative stress, oxidative DNA damage, and change of DNA methylation pattern in the liver, and these changes may contribute to the development of liver steatosis in breast cancer models.

## 1. Introduction

For over two decades, the US has experienced a rise in the proportion of overweight and obese adult population. If current trends continue, it is estimated that all adult Americans will be classified as overweight/obese by the year 2048 [1]. Similarly, many other countries are experiencing dramatic increases in obesity. Worldwide, greater than 1.9 billion adults are overweight—of which 600 million are obese [2,3]. These statistics reveal serious health implications due to the association between obesity and the risk for chronic diseases, including type 2 diabetes, cardiovascular disease, liver disease, and certain types of cancer [4]. Obese populations have higher mortality rates from all cancers and other chronic diseases [5,6,7].

Obesity is shown to have a significant impact on the metabolic profiles for a variety of cellular-tissue-organ levels in animals and humans. Several studies have shown that obesity can play an important role in the promotion of fatty liver. Fatty liver disease, ranging from simple hepatic steatosis and non-alcoholic steatohepatitis to cirrhosis, is a major health problem in the US and the world [8,9,10,11]. It is well established that liver diseases are often accompanied by significant changes in methionine cycle metabolites [12,13,14,15]. However, the full pathophysiological picture of fatty liver conditions is not completely understood and requires more research. We hypothesize that obesity will increase oxidative/nitrosative stress and DNA modification in 7,12-dimethylbenz(*α*)anthracene (DMBA)-induced mammary tumor models. Previously, we reported the methionine pathway metabolites had different serum concentration in obese rats compared to lean in obese DMBA-treated rat model [16]; however, the correlation between the serum and liver metabolic profiles has not been investigated in this model.

**Figure 1:**
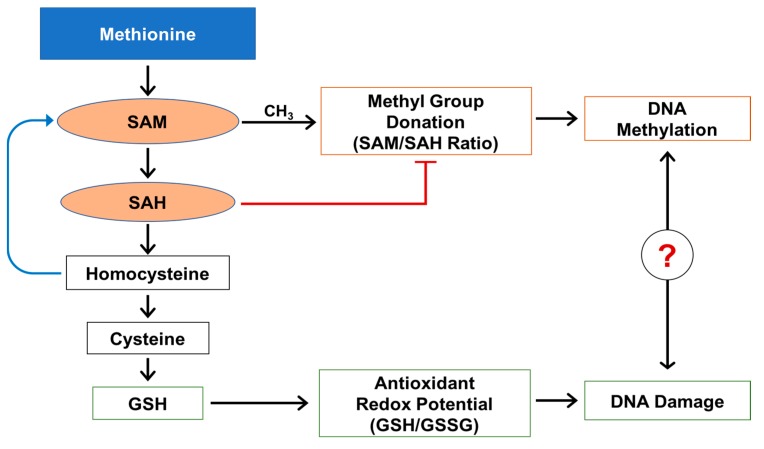
Methionine metabolism, DNA methylation, and DNA oxidative damage.

Methionine is an essential amino acid involved in multiple intracellular functions such as protein synthesis and methylation of a variety of substrates (more than 100 methylation reactions), including DNA and glutathione synthesis [17]. In the liver under physiological conditions, methionine is converted to S-adenosylmethionine (SAM), a primary source of a methyl group (CH_3_) (Figure 1). The availability of CH_3_ and activity of cell/organ specific methylases are equally important and required for transmethylation reactions. After donation of a methyl group, including DNA methylation, SAM is converted to S-adenosylhomocysteine (SAH). SAH is a very potent inhibitor of cellular methylation reactions [18,19] and is further converted to homocysteine. Homocysteine can be remethylated back to methionine using a methyl group from folate, or can be used in a downstream transsulfuration path to generate cysteine and, subsequently, glutathione.

Glutathione is the most abundant intracellular antioxidant in the liver and plays an important role in maintaining intracellular redox balance [20,21,22]. Alteration of the intracellular redox balance creates oxidative/nitrosative stress and can affect the activity of multiple intracellular proteins and affect the activity of a variety of enzymes including methylases [19,23,24].

There is a link between obesity and the development of epigenetic DNA modification and DNA damage that can lead to a chronic pathological condition in the liver. Oxidative stress is an important part of this condition as it exacerbates DNA damages and the formation of a modified base 8-OH-guanosine. 8-OH-Guanosine is well accepted and widely used as a measure for testing in the research community for monitoring and evaluating oxidative damage. DNA oxidative damage takes place frequently in cases of fatty liver and may be associated with chronic pathological conditions leading to liver fibrosis and cancer. It has been reported that fatty liver can lead to DNA damage mediated by reactive oxygen species and the formation of 8-OH-guaonosine [25,26].

**Figure 2:**
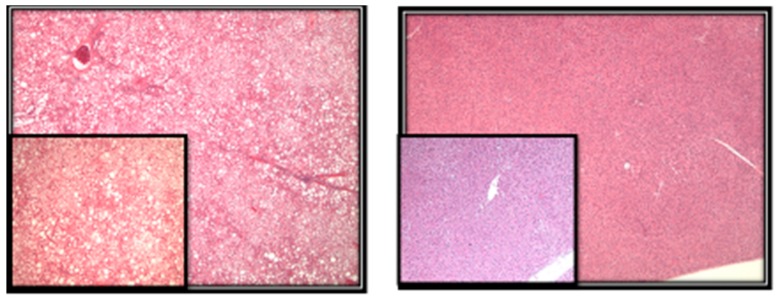
Left photomicrograph shows micro- and macro-vesicular steatosis in obese rats involving more than 75% of hepatocytes (original mag 40×, insert 100×). Right photomicrograph shows no evidence of fatty changes in lean rats (original mag 40×).

Previously, we showed that obesity increases fatty liver in a mammary tumor model [27]. The obese rats had a significantly higher steatosis score (4.90 ± 0.06) when compared to the lean rats (1.53 ± 0.11) (*p* < 0.01). Additionally, we included images that show representative hepatic liver steatosis in lean and obese rats (Figure 2). However, the effects of obesity on the level of global DNA methylation, DNA damage, and oxidative stress in liver of the DMBA-induced mammary tumors have not been reported. In the present study, we used the DMBA-induced mammary tumor obese Zucker rat model to investigate the effects of obesity on liver oxidative and nitrosative stress and on the modification of DNA bases that can contribute to the development of liver steatosis.

## 2. Results

We used a lean and obese Zucker rat model to investigate the effects of obesity on the level of global DNA methylation, DNA damage, and oxidative stress in the liver of DMBA-induced mammary tumors.

### 2.1. Methylation Circle Metabolites and Global DNA Methylation Level

**Figure 3:**
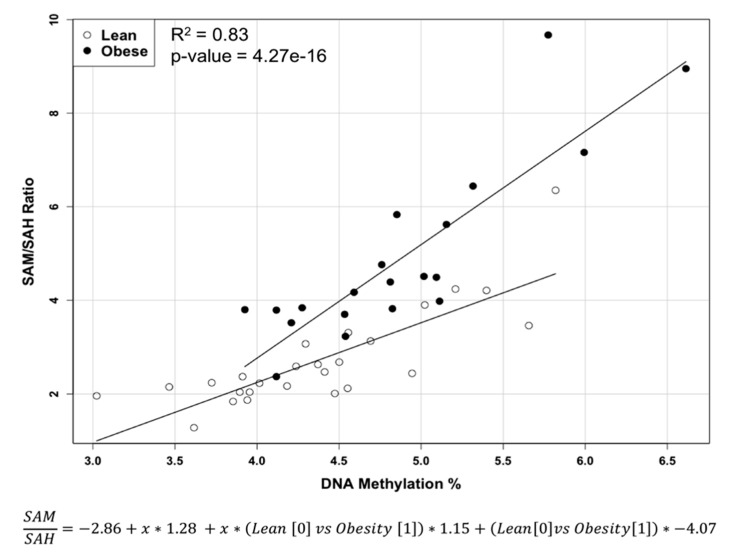
Multiple liner regression of SAM/SAH ratio and DNA methylation in liver of lean and obese Zucker rats.

Our results show (Table 1) that obesity caused significantly higher (*p* < 0.0004) methionine levels, lower (*p* < 0.0005) SAH levels, and higher (*p* < 0.0005) SAM/SAH ratios (methylation ratio). Global DNA methylation, defined as relative level of 5-methylcytosine, of obese rats was significantly (*p* < 0.02) higher compared to the lean rats. A multiple liner regression analysis of methylation ratio versus global DNA methylation shows a significant (*p*-value = 4.27 × 10^−16^) difference in linear slopes and data distribution between obese and lean animals (F(3, 42) = 67.38, *p* = 4.27 × 10^−16^, R^2^ = 0.83) (Figure 3).

### 2.2. Reduced and Oxidized Glutathione, their Ratio, and Oxidative DNA Damage Level

**Figure 4:**
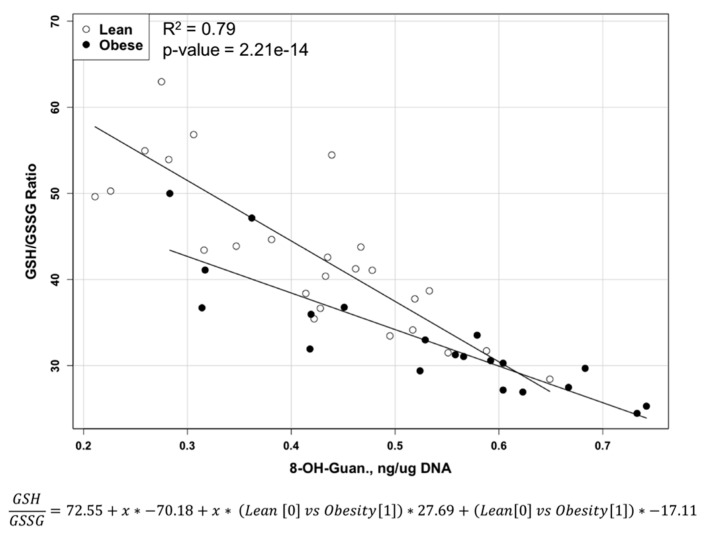
Multiple liner regression of the GSH/GSSG ratio and DNA oxidation in liver of lean and obese Zucker rats.

Levels of free reduced glutathione (GSH) (Table 2) were not significantly (*p* < 0.08) lower in obese rats compared to lean rats, but the level of free oxidized glutathione disulfide (GSSG) was significantly (*p* < 0.002) higher in obese rats compared to the lean rats. The GSH/GSSG ratio was significantly (*p* < 0.0001) lower in obese rats compared to lean rats. Obese rats developed a significantly higher (*p* < 0.004) level of oxidized guanosine (8-OH-guanosine) in DNA liver compared to lean rats. Multiple liner regression modeling of the GSH/GSSG ratio versus DNA oxidation shows significant (*p*-value = 2.21 × 10^−14^) difference in linear slope and data distribution between two obese and lean animals (F(3, 42) = 53.37, *p* = 2.21 × 10^−14^, R^2^ = 0.79) (Figure 4).

### 2.3. S-Nitrosoglutathione and 3-Nitrotyrosine Level

Levels of 3-nitrotyrosine (*p* < 0.04) as well as levels of S-nitrosoglutathione (GSNO) in obese rats were significantly higher (*p* < 0.04) compared to lean rats (Table 3).

## 3. Discussion

Our original experiment was designed to investigate the effects of obesity on mammary tumor development. We treated all rats with 7,12-dimethylbenz(α)anthracene (DMBA) and found that the liver samples were not affected by cancer in either lean or obese rats. We had an opportunity to investigate and compare liver metabolic status in these two groups of animals. In the present study, the liver of obese rats demonstrated a higher level of essential amino acid methionine. An increase in the level of methionine in the liver of obese rats was also reported by Serkova et al. [28]. This higher level of methionine could resonate and be a part of general and complicated trends of imbalance in amino acids metabolism in the livers of obese rats [29]. We were interested in investigating the effects of obesity on methionine content in the liver because almost half of human or animal body methionine metabolism happens in the liver [30] and methionine participates in biological methylation reactions and glutathione synthesis. Additionally, an increase in the level of SAM and a decrease in the SAH level in obese animals compared to the lean group resulted in higher SAM/SAH ratios in the livers of obese rats. The increase of global DNA methylation in the liver of obese rats in our study was also reported by Williams et al. [31]. The increase in the methylation ratio in the liver of an obese rat can affect global DNA methylation, which can result in hypermethylation of specific genes [32,33,34]. Additionally, despite the same line direction in multiple liner regression analysis in both groups, the slope of the liver of obese rats was significantly steeper (*p* < 10^−15^), and the data variability is different compared to lean animals. We consider global DNA hypermethylation to be an important contributor to pathogenesis of fatty liver, which can further develop to cancer [35].

In the present study, obesity led to lower levels of the reduced (active) form of glutathione, higher levels of the oxidized (inactive) form GSSG, and higher levels of oxidative stress based on lower GSH/GSSG ratios (oxidative stress ratio), as has also been previously reported by other investigators [36,37].

Increase of pro-oxidative environment contributed to an increase in DNA damage by reactive oxygen species products based on an increase in the content of oxidized guanosine (8-OH-guanosine) present. Despite the same line direction in multiple liner regression analysis in both groups, the slope of the liver of obese rats is significantly less steep (*p* < 10^−13^), and data variability is different between the two groups. This finding indicates the rate of DNA damage by reactive oxygen species will increase much faster in obese animals with the same depletion rate of the GSH/GSSG ratio. In addition to higher levels of oxidative stress in obese rats, we observed an increase in the level of nitrosative stress created by reactive nitrogen species in the livers of obese animals, as has been reported by others [38,39]. The combination of oxidative and nitrosative stress has much more potential damaging power and much higher capabilities in maintaining of chronic inflammation and immunological dysbalance in the liver [40,41,42]. The combination of oxidative and nitrosative stress with chronic inflammation conditions can be considered important contributing factors to fatty liver development in DMBA-induced mammary tumor models. The pathogenetic role of 8-OH-guanosine in the formation of fatty livers is not clear. Future study in our laboratory will focus on the role of oxidative/nitrosative stress, DNA damage, and DNA methylation status in the pathogenesis of steatosis in DMBA-induced mammary tumor models using Zucker rat models.

## 4. Materials and Methods

- *Experimental Design*: The animal protocols were approved by the Institutional Animal Care and Use Committee and the Institutional Animal Care and Use Committee of the University of Arkansas for Medical Sciences. A total of 46 five-week-old female Zucker rats (20 obese *fa/fa* and 26 lean) were obtained from Harlan Industries (Indianapolis, IN, USA). Harlan Industries performed genotyping to identify *fa/fa* and lean/lean rats at the age of 24 days. Rats were housed 2 per cage with ad libitum access to water and semi-purified diet (AIN-93G diet, Harlan Teklad, Madison, WI, USA). At 50 days of age, all rats, as part of an experiment on the effects of obesity on mammary tumor development [43], received the carcinogen 7,12-dimethylbenz(*α*)anthracene (DMBA, Sigma Chemical Co., St. Louis, MO, USA) via gavage (65 mg DMBA/kg body weight in sesame oil). Rats were euthanized at approximately 155 days later. Livers were removed and weighed. The livers were snap-frozen in liquid nitrogen and stored at −80 °C until processing for analysis of the metabolic profile related to methionine cycle and oxidative and nitrosative stress.
- *Methods*: Liver sections were evaluated for the presence of microvesicular and macrovesicular steatosis. The percentage of liver cells showing fat accumulation was estimated (Figure 2). A score of 1 to 4 was given to each section, reflecting the relative degree of steatosis in hepatocytes: 1 (<25%), 2 (25–50%), 3 (51–75%), and 4 (>75%) [27].
- To detect and quantify metabolites of our interest in the livers of Zucker rats, we used high-performance liquid chromatography with electrochemical (HPLC-ECD) and ultraviolet (HPLC-UV) detection and liquid chromatography-mass spectrometry (LC-MS) techniques. All methodological details about HPLC-ECD have been described previously [44,45]. Briefly, approximately 20 mg of frozen liver tissue were homogenized in ice-cold phosphate-buffered saline buffer. To precipitate proteins, 10% metaphosphoric acid was added to the homogenate and incubated for 30 min on ice. The samples were then centrifuged at 18,000 *g* at 4 °C for 15 min, and 20 µL or 10 µL of the resulting supernatants were injected into the HPLC or LC-MS systems accordingly for metabolite quantification. The pellet was used for protein analysis using BCA protein assay.

DNA was extracted from frozen liver samples using the Puregene DNA Purification kit (Qiagen, Valencia, CA, USA). The levels of 8-OH-guanosine and 5-methylcytosine in liver DNA were measured using HPLC-UV combined with electrospray tandem mass spectrometry (LC-MS) as previously detailed [46].

## 5. Statistical Analysis

Results are presented as mean ± standard deviation. Significant differences, *p* < 0.05, between groups were evaluated using an unpaired 2-tailed Student’s *t*-test. Multiple linear regression modeling and graphing were performed in the R statistical computing environment [47]. The linear regression modeling algorithm in the R Stats Package, stats, was used to create the multiple linear regression models as well as perform summary statistical assessment of the overall model fit and evaluation of the model coefficients. All model coefficients were significant contributors to the fitted models (*p* < 0.05).

## 6. Conclusions

In summary, obesity caused a very complex change of metabolic profile in liver with significant oxidative/nitrosative stress, oxidative DNA damage, and change of DNA methylation pattern. This combination of factors can contribute to the development of liver steatosis in breast cancer models.

Further research focus is needed to investigate the relationship between modification of DNA methylation and oxidative by DNA damage in the development of liver steatosis in breast cancer model.

## Figures and Tables

**Table 1:** Level of methionine metabolites and global DNA methylation in liver of lean and obese rats.

Metabolites	Obese (*n* = 20)	Lean (*n* = 26)	*p*
Methionine (nmol/mg protein)	0.798 ± 0.187	0.596 ± 0.129	<0.0004
SAM (nmol/mg protein)	0.868 ± 0.325	0.703 ± 0.247	0.055
SAH (nmol/mg protein)	0.184 ± 0.039	0.267 ± 0.069	<0.0005
SAM/SAH	4.91 ± 1.882	2.72 ± 1.051	<0.0005
5-methylcytosine (%)	4.882 ± 0.675	4.37 ± 0.673	<0.02

mean ± standard deviation.

**Table 2:** Level of glutathione metabolites and DNA oxidation in liver of lean and obese rats.

Metabolites	Obese (*n* = 20)	Lean (*n* = 26)	*p*
GSH (nmol/mg protein)	28.2 ± 6.28	31.8 ± 6.96	<0.08
GSSG (nmol/mg protein)	0.864 ± 0.157	0.738 ± 0.125	<0.002
GSH/GSSG	32.9 ± 6.77	43.8 ± 10.13	<0.0001
8-OH-Guanosine (ng/µg DNA)	0.528 ± 0.139	0.409 ± 0.121	<0.004

mean ± standard deviation.

**Table 3:** Level of nitrosative stress metabolites in liver of lean and obese rats.

Metabolites	Obese (*n* = 20)	Lean (*n* = 26)	*p*
GSNO (pmol/mg protein)	37.2 ± 7.46	30.4 ± 12.51	<0.04
nitrotyrosine (nmol/mg protein)	0.261 ± 0.035	0.239 ± 0.036	<0.04

mean ± standard deviation.

## List of Abbreviations

BCA	bicinchoninic acid
C	Celsius
CH_3_	methyl group
DMBA	7,12-dimethylbenz(α)anthracene
DNA	deoxyribonucleic acid
*Fa*	fatty
GSH	glutathione
GSNO	S-nitrosoglutathione
GSSG	glutathione disulfide
HPLC	high-performance liquid chromatography
HPLC-ECD	high-performance liquid chromatography with electrochemical detection
HPLC-UV	high-performance liquid chromatography with ultraviolet detection
LC-MS	liquid chromatography-mass spectrometry
Mg	milligram
*n*	number
ng	nanogram
nmol	nanomole
*p*	*p*-value
pmol	picomole
SAH	S-adenosylhomocysteine
SAM	S-adenosylmethionine
SD	standard deviation
Mg	microgram
µL	microliter
